# Endothelial Cell Activation by SARS-CoV-2 Spike S1 Protein: A Crosstalk between Endothelium and Innate Immune Cells

**DOI:** 10.3390/biomedicines9091220

**Published:** 2021-09-14

**Authors:** Bianca Maria Rotoli, Amelia Barilli, Rossana Visigalli, Francesca Ferrari, Valeria Dall’Asta

**Affiliations:** Laboratory of General Pathology, Department of Medicine and Surgery, University of Parma, 43125 Parma, Italy; biancamaria.rotoli@unipr.it (B.M.R.); amelia.barilli@unipr.it (A.B.); rossana.visigalli@unipr.it (R.V.); francesca.ferrari@unipr.it (F.F.)

**Keywords:** COVID-19, spike S1 protein, endothelial activation, macrophages, cytokines

## Abstract

Background. Emerging evidences suggest that in severe COVID-19, multi-organ failure is associated with a hyperinflammatory state (the so-called “cytokine storm”) in combination with the development of a prothrombotic state. The central role of endothelial dysfunction in the pathogenesis of the disease is to date accepted, but the precise mechanisms underlying the associated coagulopathy remain unclear. Whether the alterations in vascular homeostasis directly depend upon the SARS-CoV-2 infection of endothelial cells or, rather, occur secondarily to the activation of the inflammatory response is still a matter of debate. Here, we address the effect of the SARS-CoV-2 spike S1 protein on the activation of human lung microvascular endothelial cells (HLMVEC). In particular, the existence of an endothelium-macrophage crosstalk in the response to the spike protein has been explored. Methods and Results. The effect of the spike protein is addressed in human lung microvascular endothelial cells (HLMVEC), either directly or after incubation with a conditioned medium (CM) of human monocyte-derived macrophages (MDM) previously activated by the spike S1 protein (CM-MDM). Both MDM and HLMVEC are activated in response to the S1 protein, with an increased expression of pro-inflammatory mediators. However, when HLMVEC are exposed to CM-MDM, an enhanced cell activation occurs in terms of the expression of adhesion molecules, pro-coagulant markers, and chemokines. Under this experimental condition, ICAM-1 and VCAM-1, the chemokines CXCL8/IL-8, CCL2/MCP1, and CXCL10/IP-10 as well as the protein tissue factor (TF) are markedly induced. Instead, a decrease of thrombomodulin (THBD) is observed. Conclusion. Our data suggest that pro-inflammatory mediators released by spike-activated macrophages amplify the activation of endothelial cells, likely contributing to the impairment of vascular integrity and to the development of a pro-coagulative endothelium.

## 1. Introduction

Coronavirus disease-2019 (COVID-19) is a highly infectious respiratory syndrome caused by the new coronavirus SARS-CoV-2 (Severe Acute Respiratory Syndrome Coronavirus 2) [1,2]. The latter is an RNA virus that targets host cells through the interaction of the spike S1 glycoprotein with the angiotensin-converting enzyme 2 (ACE2) [3]. In most cases, the clinical spectrum of COVID-19 ranges from asymptomatic to a mild-to-moderate, self-limiting disease, with signs including fever, dry cough, fatigue, diarrhea, conjunctivitis, alterations in taste and smell, and pneumonia [4]; in some patients, however, the disease can progress to severe conditions that may ultimately lead to a fatal outcome [5]. Among these, respiratory insufficiency due to the acute respiratory distress syndrome (ARDS) is the most life-threatening condition; however, multiple organ failure referable to the activation of coagulation and thrombosis, or to disseminated intravascular coagulation (DIC) also represent common causes of death in COVID-19 patients [6]. The severity of the disease is strictly associated with an imbalance in innate immune responses and the manifestation of a hyperinflammatory state (the so-called “cytokine storm”), with high plasma levels of C-reactive protein, IL-6, and other proinflammatory cytokines in circulation [7].

Although pneumocytes are the primary targets of SARS-CoV-2, the central role of endothelial dysfunction in the pathogenesis of severe COVID-19 is to date accepted worldwide [8]. Whether the vast alterations in vascular homeostasis directly depend upon SARS-CoV-2 infection of endothelial cells or, rather, occur secondarily to the activation of the inflammatory response is still a matter of debate; however, the most convincing pathogenic model to date includes the simultaneous involvement of both mechanisms [9]. As for the ability of the virus to infect endothelial cells directly, the question is still controversial. Indeed, while clinical evidences and post-mortem studies have provided evidences of SARS-CoV-2 entry into the cells [4,10], only very low levels up to the absence of the ACE2 receptor have been detected in the endothelium [11]. In this regard, however, several cell-surface receptors other than the ACE2 have been shown to mediate viral entry into the cells [12,13]; moreover, co-receptors such as neuropilin-1 (NRP1) and neuropilin-2 (NRP-2) are of a certain relevance in the infectivity of Sars-Cov-2 [14]. Neuropilin-1, in particular, is essential for vascular development and the maintenance of endothelial homeostatic function, and promotes physiological and pathological angiogenesis [15]; furthermore, it has been shown to regulate the endothelium-dependent inflammatory responses at the level of the blood–brain barrier [16]. Both NRP-1 and NRP-2 are abundantly expressed in pulmonary and olfactory endothelial cells [17,18,19]; thus, the hypothesis of a direct interaction of the virus with the endothelium is nevertheless reasonable.

On the other hand, it has been postulated that also immune-mediated mechanisms may also be involved in the emergence of COVID19-associated endothelial dysfunctions [20,21,22]; an experimental validation of this hypothesis, however, is still needed.

In this context, the aim of the present study was to define the effects of the SARS-CoV-2 spike S1 protein on the activation of human endothelial cells, either induced directly by the viral protein or mediated by S1-treated macrophages, so as to explore the possible involvement of immune-mediated mechanisms.

## 2. Materials and Methods

### 2.1. Cell Culture

Human monocytes were isolated from buffy coats of healthy donors, screened for viral infections, and provided by the Unit of Immunohematology and Transfusion of the Azienda Ospedaliero-Universitaria of Parma, as previously described [23]. The protocol of the study was approved by the local ethical committee (protocol 43899 del 16 December 2015) and conducted in accordance with the principles of the Declaration of Helsinki (1964). Briefly, buffy coats were diluted 1:4 with PBS and layered onto 15 mL Lympholyte-H (Euroclone, Milan, Italy) before being centrifuged at 800 g for 20 min at 20 °C. PBMCs at the interface were collected, washed twice with PBS, then suspended in the RPMI1640 medium supplemented with 10% endotoxin-free fetal bovine serum (FBS) and seeded on plasticware. After a 30-minute incubation at 37 °C in an atmosphere with 5% CO_2_, non-adherent cells were removed with 3 vigorous washes in prewarmed RPMI1640. Monocyte-derived macrophages (MDM) were obtained by incubating adherent monocytes for 5 d in the RPMI1640 medium supplemented with 10% endotoxin-free fetal bovine serum (FBS) and 50 ng/mL of recombinant human granulocyte Mϕ colony-stimulating factor (GM-CSF, ReliaTech, Lower Saxony, Germany). Human lung microvascular endothelial cells (HLMVEC) were purchased from Neobiotech (Pasadena, CA, USA); cells were routinely grown according to the manufacturer’s instructions and employed between passages 1 and 6.

For the experiments, both MDM and endothelial cells were treated with the SARS-CoV-2 spike recombinant protein (S1 subunit; ARG70218; Arigo Biolaboratories, Hsinchu City, Taiwan), whose endotoxin contamination is <0.1 EU/µg of protein, as certified by the manufacturer; where indicated, His-tagged recombinant albumin (Human Serum Albumin Protein, His Tag; HSA-H5220; Acrobiosystems, Newark, DE, USA) was added to the cell culture as negative control. To exclude any interference by lipopolysaccharide (LPS) in the effects addressed, the S1 protein was mixed with Polymyxin B (Merck, Monza, Italy), an antibiotic employed as an LPS inhibitor, and incubated for 30 min at RT before the addition to the cell cultures; for the same reason, when required, cell monolayers were pre-incubated with 5 µg/mL TLR4-neutralizing antibody (Cayman Chemicals, Firenze, Italy) for 30 min before the addition of the S1 solution. Where indicated, the incubation medium of MDM maintained for 16 h in the absence or presence of 10 nM S1 supplemented with 2 µg/mL Polymyxin B, was collected and employed as the conditioned medium (MDM-CM) for the treatment of HLMVEC.

### 2.2. RT-qPCR Analysis

Gene expression was analyzed through RT-qPCR, as previously described [24]. A total of 1µg of cDNA was obtained upon reverse transcription of the total RNA with the RevertAid First Strand cDNA Synthesis Kit (Thermo Fisher Scientific, Monza, Italy); qPCR was then performed on a StepOnePlus Real-Time PCR System (Thermo Fisher Scientific) by employing specific forward/reverse primer pairs (Table 1. References are indicated for previously validated pairs; where absent, refer to Figure S1 for validation) and SYBR™ Green or TaqMan Gene Expression Master Mix (Thermo Fisher Scientific). The absolute quantification of the mRNA of genes coding for the SARS-CoV-2 spike receptor (angiotensin-converting enzyme 2; ACE2) and co-receptors (Neuropilin 1 and 2; NRP1 and NRP2) was performed and the number of molecules for each mRNA was calculated after normalization for the housekeeping gene (ribosomal-like protein 15, RPL15, Gene ID:6138) as described previously [25]. The effect of the spike S1 protein on gene expression was instead determined with the ∆∆Ct method [26] and shown, relative to RPL15, as fold change of untreated control cells (=1).

### 2.3. Cytokine Analysis

The amount of cytokines released in the culture medium by treated cells was quantified by employing the Cytokine Human Magnetic 10-Plex Panel (Thermo Fisher Scientific) for Luminex™ platform, as already described [28].

### 2.4. Western Blot Analysis

For the determination of protein expression, cells were lysed in RIPA buffer supplemented with a cocktail of protease inhibitors (cOmplete™, Mini, EDTA-free, Roche, Basel, Switzerland). Western Blot analysis was performed, as previously described [27]. Briefly, 20 µg of proteins was separated on SDS-PAGE (Bolt™ 4–12% Bis-Tris mini protein gel, Thermo Fisher Scientific) and electrophoretically transferred to PVDF membranes (Immobilon-P membrane, Merck). Membranes were incubated for 1 h at RT in Tris-buffered saline solution (TBS; 50 mM Tris-HCl pH 7.5, 150 mM NaCl) containing 5% non-fat dried milk, then incubated overnight at 4 °C in TBST (TBS + 0.5% Tween) supplemented with 5% BSA and anti-ICAM-1, anti-VCAM-1, or anti-tissue factor (TF) purified rabbit polyclonal antibodies or anti-CD31/PECAM mouse monoclonal antibody (1:2000, Cell Signaling Technology, Beverly, MA, USA). Anti-vinculin (Merck) or anti-β-actin (Cell Signaling Technology) mouse monoclonal antibodies (1:2000) were employed as the internal standard. Immunoreactivity was visualized with SuperSignal™ West Pico PLUS Chemiluminescent HRP Substrate (Thermo Fisher Scientific). Western Blot images were captured with the iBright FL1500 Imaging System (Thermo Fisher Scientific) and analyzed with the iBright Analysis Software.

### 2.5. Statistical Analysis

GraphPad Prism 9 (GraphPad Software, San Diego, CA, USA) was used for statistical analysis. *p* values were calculated with a two-tailed Student’s *t*-test for unpaired data or with “one-sample *t*-test”. *p* < 0.05 was considered significant.

### 2.6. Materials

Endotoxin-free fetal bovine serum (South America origin; EU Approved) was purchased from EuroClone (Italy), and GM-CSF (ReliaTech) from Vinci-Biochem (Florence, Italy). Merck (Italy) was the source of all other chemicals, unless otherwise specified.

## 3. Results

**Figure 1:**
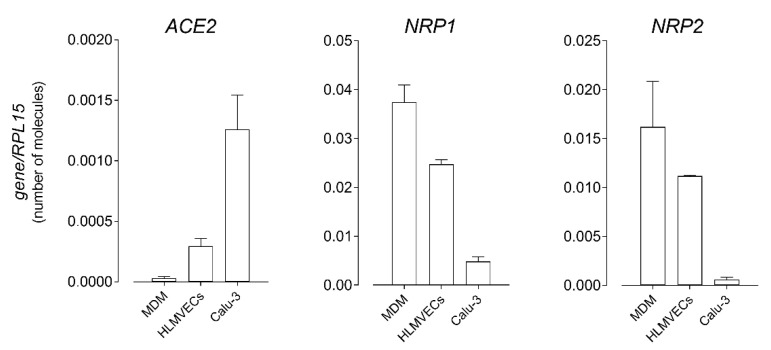
The absolute number of mRNA molecules for the indicated genes was calculated in human monocyte-derived macrophages (MDM), in human lung microvascular endothelial cells (HLMVEC), and in Calu-3 respiratory epithelial cells by means of RT-qPCR and expressed relative to that of the housekeeping gene RPL15, as described in Materials and Methods. Data are means ± SEM (Standard Error of Measurement) of six (MDM) or three (HLMVEC and Calu-3) determinations, each performed in duplicate.

Since the presence of the angiotensin-converting enzyme 2 (ACE2), target of the spike S1, is still controversial in some tissues, we first verified the expression of the enzyme in monocyte-derived macrophages (MDM) and human lung microvascular endothelial cells (HLMVEC); Calu-3 respiratory epithelial cells, which are known to express the receptor on the apical membrane [29], were employed for comparison. As shown in Figure 1, ACE2 was detected in all the models tested, although mRNA levels were much lower in both MDM and HLMVEC than in Calu-3. Instead, the transcripts for the neuropilins NRP1 and NRP2, co-receptors potentiating the infectivity of SARS-CoV-2 [17], were much more abundant in macrophages and endothelial cells than in respiratory epithelium. Although expressed to varying degrees, the detection of viral entry factors in MDM and HLMVEC is at least consistent with the direct susceptibility of these cells to SARS-CoV-2 infection; thus, we next addressed the effects of the spike S1 protein on the phenotype and function of both macrophages and endothelial cells.

**Figure 2:**
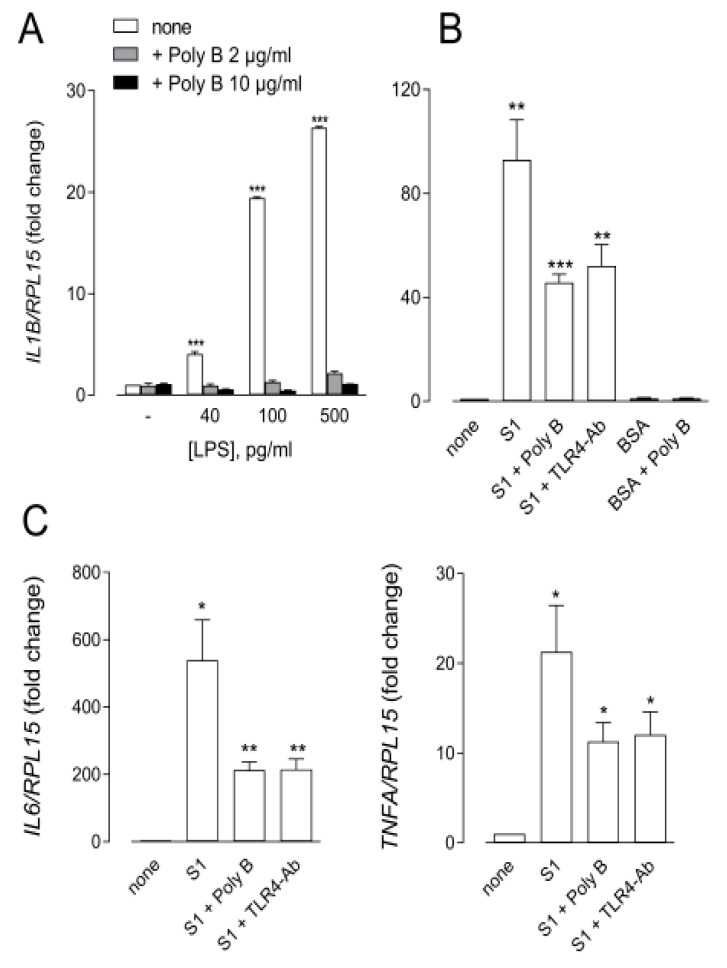
MDM were incubated for 6 h in the absence (none) or in the presence of the indicated concentrations of LPS, either alone or supplemented with 2 or 10 µg/mL Polymyxin B (Poly B) (Panel **A**), or in the presence of 10 nM SARS-CoV-2 spike (S1) or His-tagged albumin (BSA) employed alone or combined with 2 µg/mL Poly B or 5 µg/mL TLR4 neutralizing antibody (TLR4-Ab), as indicated (Panels **B**,**C**). The expression of the indicated genes was then measured by means of RT-qPCR, normalized for that of the housekeeping gene RPL15, and expressed relative to untreated control cells (=1). Data are means ± SEM of four experiments, each performed in duplicate. * *p* < 0.05, ** *p* < 0.01, *** *p* < 0.001 vs. untreated cells with “one-sample *t*-test”.

As far as the innate immune system is concerned, it must be underlined that a synergism has recently been described in macrophages between lipopolysaccharide (LPS) and the S1 protein, leading to a boosting of pro-inflammatory actions in vitro and in vivo [30]. Therefore, to specifically define the effects of spike in these cells, it is mandatory to first ensure the removal of any LPS contamination from the protein. To this end, and for our study, we employed an S1 protein with certified low endotoxin content (<0.1 EU/µg protein); after further testing with the Pierce™ Chromogenic Endotoxin Quant kit (Thermo Fisher), this actually showed a contamination by LPS roughly corresponding to 30–50 pg/µg of the protein. Then, to avoid any possible interference by the endotoxin, we decided to mix S1 with Polymyxin B, a potent LPS-binding nonapeptide employed in clinical practice as an antibiotic [31]. In this regard, a preliminary experiment was performed to validate the efficacy of Polymyxin B in preventing the induction of IL1β transcript by increasing the concentrations of LPS in human MDM (Figure 2, Panel A). Since the results obtained demonstrated that 2 µg/mL Polymyxin B almost completely abolished the effects of LPS up to 500 pg/mL, this concentration of the antibiotic was thereafter employed under our experimental conditions so as to avoid any interference by LPS in the effects observed. As shown in Figure 2, Panels B and C, the incubation of human MDM for 6 h with 10 nM spike alone (roughly containing 40 pg/mL LPS) markedly induced the expression of genes for IL-1β, IL-6, and TNFα; conversely, the treatment with 10 nM His-tagged albumin (BSA), employed as a negative control, had no effect. The simultaneous addition of Polymyxin B, although decreasing the effect of S1, still significantly induced the expression of the cytokines. A similar stimulation remained evident when MDM were preincubated with a neutralizing antibody targeting the LPS receptor TLR4 (TLR4-Ab). Taken together, these results, besides confirming that the contamination of the spike protein by endotoxin actually boosts the inflammatory response of human macrophages, definitively demonstrate that the S1 protein is able *per se* to induce an inflammatory phenotype via a TLR4-independent pathway in MDM.

**Figure 3:**
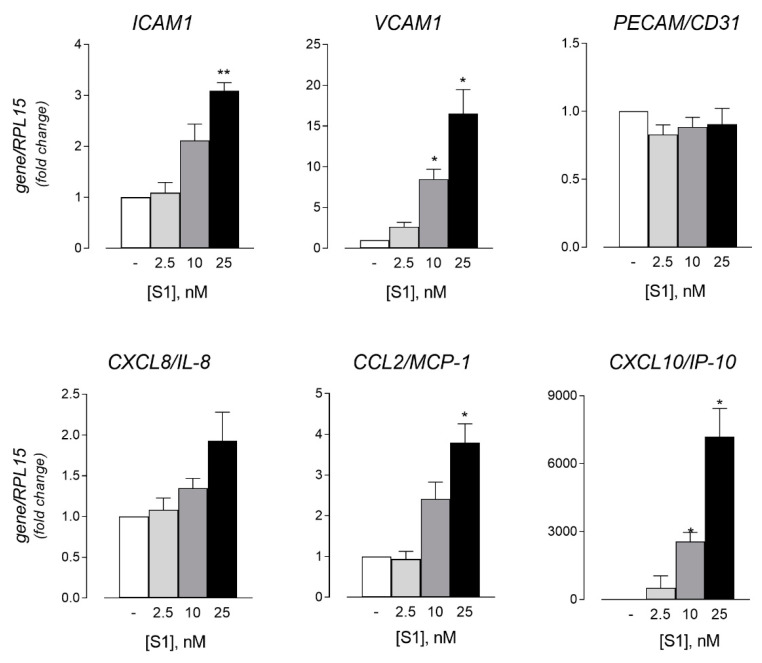
HLMVEC were incubated for 6 h in the absence (-) or in the presence of the indicated concentrations of SARS-CoV-2 spike protein supplemented with 2 µg/mL Polymyxin B (S1). The expression of the indicated genes was then measured by means of RT-qPCR, normalized for that of the housekeeping gene RPL15, and expressed relative to untreated control cells (=1). Data are means ± SEM of three experiments, each performed in duplicate. * *p* < 0.05, ** *p* < 0.01 vs. none (-) with “one-sample *t*-test”.

The effect of the spike protein was next addressed in human lung microvascular endothelial cells (HLMVEC). To this end, cells were incubated for 6 h in the presence of increasing concentrations (from 0.25 to 25 nM) of S1 supplemented with 2 µg/mL Polymyxin B, and the endothelial activation was then monitored in terms of the expression of adhesion molecules. As indicated in Figure 3, the mRNA levels of both immunoglobulin superfamily members ICAM-1 and VCAM-1 were induced by the spike in a dose-dependent manner, while the expression of PECAM1/CD31 was not modified by the incubation with the protein at any concentration. Under the same conditions, a slight increase was also observed for the expression of the chemokines CXCL8/IL-8, and, even more markedly, for CCL2/MCP-1 (monocyte chemoattractant protein-1) and CXCL10/IP-10 (IFN-γ-inducible protein 10); for the latter, the induction was particularly impressive due to the very low level of expression in untreated cells.

**Figure 4:**
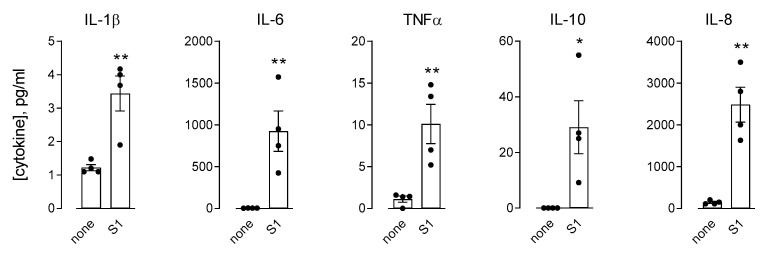
MDM were incubated for 16 h in the absence (none) or in the presence of 10 nM of SARS-CoV-2 spike protein supplemented with 2 µg/mL Polymyxin B (S1). The amount of the indicated cytokines was measured with an ELISA assay, as described in Materials and Methods. Data are means ± SEM of four independent determinations, each represented by the different dots. * *p* < 0.05, ** *p* < 0.01 vs. none, with a two-tailed Student’s *t*-test for unpaired data.

**Figure 5:**
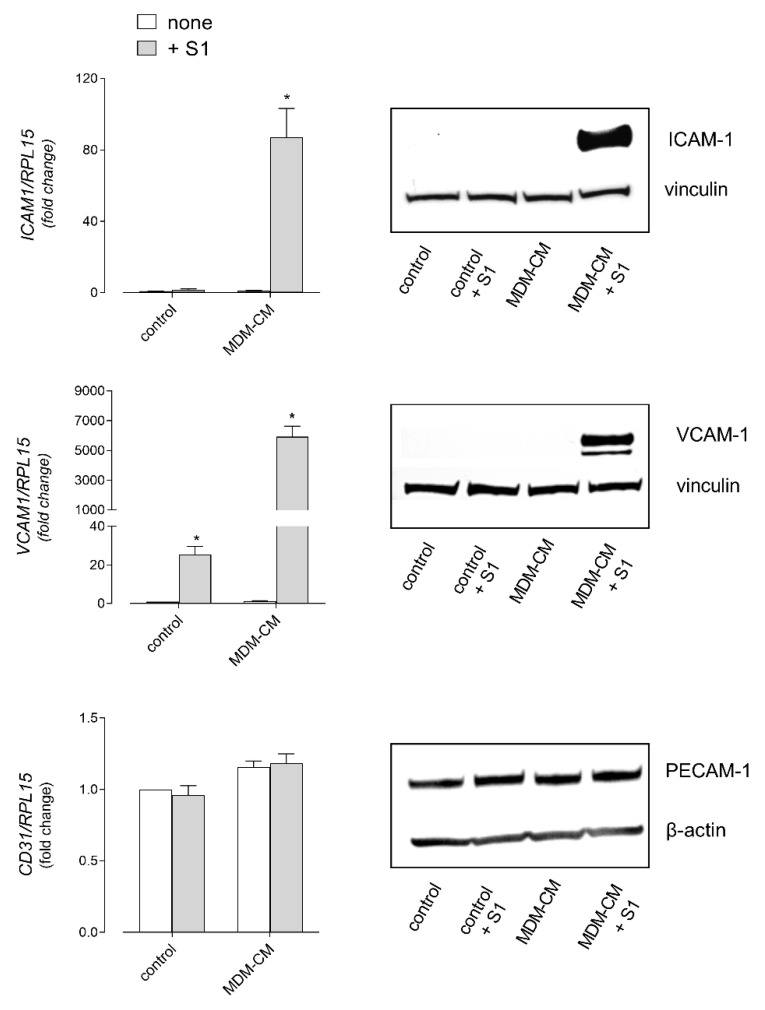
HLMVEC were incubated for 6 h in fresh RPMI1640 growth medium (control) in the absence (none) or in the presence of 10 nM SARS-CoV-2 spike protein supplemented with 2 µg/mL Polymixin B (S1) or in MDM conditioned medium (MDM-CM), obtained by incubating MDM for 16 h in the absence (none) or in the presence of 10 nM S1 and 2 µg/mL Polymyxin B. The expression of the indicated genes (**left** panels) was then measured by means of RT-qPCR, normalized for that of the housekeeping gene RPL15, and expressed relative to none in control cells (=1); data are means ± SEM of three experiments, each performed in duplicate. * *p* < 0.05 vs. none in control with “one-sample *t*-test”. The amount of the corresponding proteins (**right** panels) was assessed by means of Western Blot analysis (see Section 2); the representative blots shown were repeated twice and gave comparable results.

**Figure 6:**
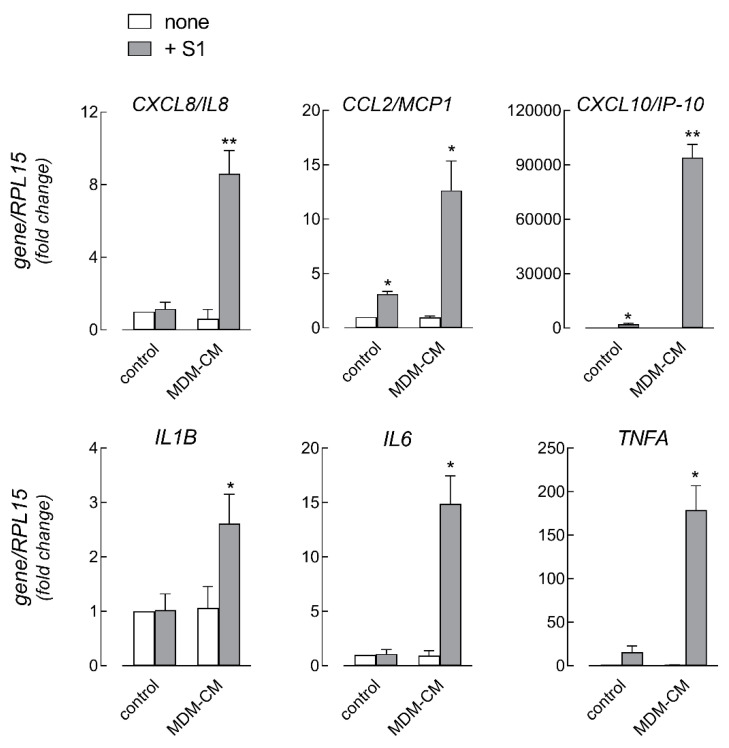
HLMVEC were incubated for 6 h in fresh RPMI1640 (control) or in MDM-conditioned medium (MDM-CM), as described in Figure 5. The expression of the indicated genes was then measured by means of RT-qPCR, normalized for that of the housekeeping gene RPL15, and expressed relative to none in control cells (=1). Data are means ± SEM of three experiments, each performed in duplicate. * *p* < 0.05, ** *p* < 0.01 vs. none in control with “one-sample *t*-test”.

Next, to address the role of immune cells in strengthening the effects of the spike on endothelial cells, we set up experiments based on the treatment of HLMVEC with the conditioned medium (CM) from human MDM (CM-MDM) previously incubated for 16 h with 10 nM S1 protein. To this end, we first analyzed the effects of S1 treatment on macrophages in terms of cytokine secretion (Figure 4). In line with the analysis of the gene expression (see Figure 2), the amount of IL-1β, IL-6, and TNFα was significantly increased upon exposure to the spike. Under the same conditions, the concentration of IL-8 and IL-10 was also higher than in the CM from untreated control cells, while the levels of IL-2, IL-4, IL-5, and IFNγ remained beyond the limit of detection of the assay (not shown). Endothelial cells were then incubated for 6 h in the presence of macrophage-conditioned medium (MDM-CM), so as to evaluate the immune-mediated effects of the spike protein on endothelial activation. As for the expression of adhesion molecules (Figure 5), the mRNA levels of ICAM-1 and VCAM-1, already increased by the treatment with 10 nM S1 alone (see Figure 3 for comparison), were dramatically induced upon incubation with CM from spike-treated macrophages. Consistently, the corresponding proteins, not even detectable in S1-treated cells, were readily evident in HLMVEC maintained in the presence of spike-containing MDM-CM. However, no difference was observed in the expression of PECAM1/CD31. Under the same conditions, the expression of chemokines CXCL8/IL-8, CCL2/MCP1, and CXCL10/IP-10 was also further induced with respect to cells treated with spike alone (Figure 3 and Figure 6, for comparison); similarly, the mRNAs encoding the cytokines IL-1β and, to a greater extent, IL-6 and TNFα were more abundant than in S1-treated control cells (Figure 6).

**Figure 7:**
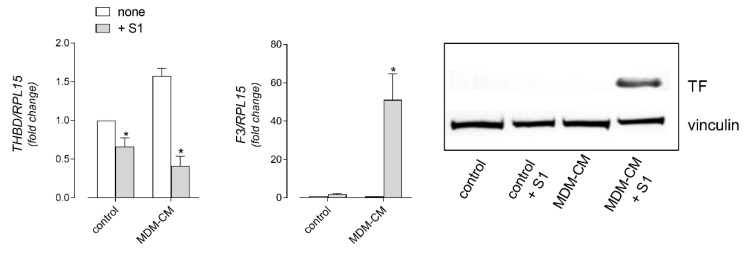
HLMVEC were incubated for 6 h in fresh RPMI1640 (control) or in MDM conditioned medium (MDM-CM), as described in Figure 5. The expression of the indicated genes (**left** and **middle** panels) was then measured by means of RT-qPCR, normalized for that of the housekeeping gene RPL15, and expressed relative to none in control cells (=1); data are means ± SEM of three experiments, each performed in duplicate. * *p* < 0.05, vs. none in control, with “one-sample *t*-test”. The amount of the tissue factor (TF) protein was assessed by means of Western Blot analysis, as described in Materials and Methods (**right** panel); the representative blot shown was repeated twice and gave comparable results.

Lastly, the effects of the SARS-CoV-2 spike S1 protein on the endothelial phenotype were evaluated in terms of the expression of genes for pro-coagulant markers, namely thrombomodulin (THBD), a major endothelial cell anticoagulant, and the thrombogenic protein tissue factor (F3/TF). Interestingly, a modest decrease of THBD expression and a concomitant slight induction of F3 mRNA were observed in cells treated with 10 nM S1 alone (Figure 7); both effects were, however, much more evident and reached statistical significance upon the incubation of HLMVEC with the MDM-CM from the spike-treated cells, when the pro-coagulant TF was detectable at the protein level (Figure 7).

## 4. Discussion

Increasing evidence indicates that the loss of vascular integrity and the development of a pro-coagulative endothelium are involved in the initiation and propagation of acute respiratory distress syndrome (ARDS) in COVID-19 through the induction of endotheliitis and inflammatory cell infiltration in the lungs [22]. Accordingly, severe COVID-19 is now considered a microvascular disorder, in which endothelial dysfunction plays a pivotal role [32,33]; in this context, the endothelium appears both as an effector that contributes to inflammation and thrombosis, and as a target organ, whose dysfunction may contribute to poor outcome [22,34,35,36,37]. Although the involvement of virus-dependent and immune-mediated mechanisms has been widely postulated [9], the molecular and cellular pathways specifically responsible for the impairment of endothelial integrity in COVID-19 remain poorly defined thus far. In this regard, the aim of our study was to investigate the response of the endothelium to SARS-CoV-2 by addressing the activation of human lung microvascular endothelial cells (HLMVEC) directly induced by the viral spike S1 protein or mediated by S1-treated macrophages.

The results we obtained confirmed the presence of transcripts for both NRP1 and NRP2 co-receptors in HLMVEC as well as in monocyte-derived macrophages (MDM). In the same cells, ACE2 mRNA was also present, albeit at much lower levels than in Calu-3 respiratory epithelial cells, hence indicating that both models are plausible direct targets of the virus. Although our study does not demonstrate an effective interaction between the spike S1 protein and NRP1, the role of this co-receptor in potentially facilitating SARS-CoV-2 entry into endothelial cells deserves to be further investigated.

The incubation of HLMVEC with the S1 protein significantly induced the expression of typical activation markers such as adhesion molecules ICAM-1 and VCAM-1, and inflammatory chemokines CCL2/MCP-1 and CXCL10/IP-10. Similar effects have been described in the human brain and in pulmonary artery endothelial cells, where a spike-dependent induction of the pro-inflammatory response and signs of cell damage have been observed [38,39]. Moreover, infection with SARS-CoV-2 caused significant changes in the expression of genes that regulate coagulation and inflammation in pulmonary microvascular endothelial cells transduced with the recombinant ACE2 receptor [40]. More recently, Raghavan et al. showed that the spike S1 causes the degradation of endothelial junctional proteins in primary brain microvascular endothelial cells of mice, thus affecting the endothelial barrier function [41]. Taken together, these findings sustain the hypothesis of a direct interaction of the virus with the endothelium.

Interestingly, the activation we observed upon the exposure of endothelial cells to the spike protein was dramatically strengthened when HLMVEC were incubated in the presence of the conditioned medium from S1-treated monocyte-derived macrophages (CM-MDM); we can thus conclude that, aside from the direct effect of the virus, immune-mediated mechanisms may also contribute to endothelial dysfunction during SARS-COV2 infection. According to our and others’ data [22], the most plausible reason for the immune-dependent strengthening of the endothelial response is an increased secretion of pro-inflammatory mediators by spike-treated monocytes/macrophages, which in turn may stimulate endothelial cells. In our study, the incubation of MDM with the S1 protein produced a massive release of cytokines and chemokines, namely IL-1β, IL-6, TNFα, IL-8, and IL-10. Similar results were described by Karwaciak et al. in GM-CSF differentiated human macrophages [42] and bone marrow-derived mice macrophages [43]. Collectively, these findings are consistent with the elevated plasma levels of inflammatory markers observed in COVID-19 patients [44,45], where the profile of IL-6, CCL2/MCP-1, and CXCL10/IP-10 are associated with the severity of the disease [46]. For IL-6 in particular, our results highlighted an impressive secretion of the cytokine by S1-treated macrophages in vitro, as compared to the modest release of IL1β and TNFα. This finding is in line with the elevated levels of IL-6, but not IL-1β, found in the sera of patients with a severe form of the disease [47].

The molecular mechanism underlying the induction of pro-inflammatory mediators by the SARS-COV-2 S1 protein was attributed to the TLR4-dependent activation of NF-κB and the stress-dependent mitogen-activated protein kinase (MAPK) signaling pathways in murine peritoneal macrophages and human THP-1-derived macrophages [48]. Conversely, our results exclude the involvement of this receptor in S1 signaling, since the addition of a TLR4-neutralizing antibody actually lowers but does not abolish the inflammatory response of human macrophages. Whether this discrepancy is due to the different cell models employed in the two studies or, rather, to the interference by LPS contamination in the experiments by Shirato et al. remains to be verified. Certainly, our data demonstrate that particular attention must be paid to endotoxin contamination when employing commercial S1 proteins in order to properly discriminate the effects of the spike from those dependent on the contaminant LPS.

Lastly, a distinctive feature of COVID-19 lies in the coagulation/fibrinolytic abnormalities defined as elevated plasma levels of D-dimer, factor VIII, and von Willebrand factor [49]. In this regard, a self-amplifying cycle of excessive inflammation was proposed to be a primary cause of the impairment of the anti-thrombogenic properties of the vascular endothelium in COVID-19, which ultimately leads to the formation of thrombi in the vessels [11]. In particular, an upregulation of the endothelial tissue factor (TF) has been assumed to play a central role in the pathogenesis of the thrombotic complications of COVID-19 [50,51,52], although a definite demonstration still lacks. Here, we demonstrated that the incubation of HLMVEC with cytokines released by spike-treated macrophages actually causes a huge increase in the expression of the tissue factor (TF), undetectable under control conditions and a concomitant significant decrease in the expression of the anti-thrombotic thrombomodulin. Our findings are in line with the recent study by Francischetti et al., which illustrates a normal expression of thrombomodulin in the alveolar capillaries of control cases and the loss of the protein in COVID-19 patients [53]; as suggested by the authors, an upregulation of pulmonary TF with the loss of thrombomodulin emerge as a potential link to immunothrombosis in COVID-19.

Taken together, our findings highlight the relevance of innate immune cells in the spike-dependent induction of a pro-coagulative phenotype in the endothelium and provide a pathogenetic model for endothelial dysfunction in COVID-19 based on a crosstalk between immune and endothelial cells, both targets of the spike S1 protein. From this perspective, inflammatory cytokines secreted by macrophages are expected to amplify the activation of endothelial cells that, in turn, produce chemokines that likely recruit circulating monocytes; the onset of this positive loop may account for the dysregulation of inflammatory responses.

## Figures and Tables

**Table 1:** Sequence of primer pairs employed for RT-qPCR analysis.

Gene/Protein Name (Gene ID)	Forward Primer	Reverse Primer
*RPL15*/RPL15 (6138)	Hs03855120_g1 (TaqMan^®^ Assay, Thermo Fisher Scientific)	
*ACE2*/ACE2 (59272)	ACAGTCCACACTTGCCCAAAT	TGAGAGCACTGAAGACCCATT
*NRP1*/NRP1 (8829)	ACCCAAGTGAAAAATGCGAATG	CCTCCAAATCGAAGTGAGGGTT
*NRP2*/NRP2 (8828)	GCTGGCTATATCACCTCTCCC	TCTCGATTTCAAAGTGAGGGTTG
*IL1B*/IL-1β (3553)	Hs99999029_m1 (TaqMan^®^ Assay, Thermo Fisher Scientific)	
*IL6*/IL-6 [27] (3569)	AACCTGAACCTTCCAAAGATGG	TCTGGCTTGTTCCTCACTACT
*CXCL8*/IL-8 [27] (3576)	ACTGAGAGTGATTGAGAGTGGAC	AACCCTCTGCACCCAGTTTTC
*CXCL10*/IP-10 (3627)	GTGGCATTCAAGGAGTACCTC	TGATGGCCTTCGATTCTGGATT
*CCL2*/MCP1 [28] (6347)	CAGCCAGATGCAATCAATGCC	TGGAATCCTGAACCCACTTCT
*TNFA*/TNFα [28] (7124)	ATGAGCACTGAAAGCATGATCC	GAGGGCTGATTAGAGAGAGGTC
*ICAM1*/ICAM-1 (3383)	TGAACCCCACAGTCACCTATG	CTCGTCCTCTGCGGTCAC
*VCAM1*/VCAM-1 (7412)	GGGAAGATGGTCGTGATCCTT	TCTGGGGTGGTCTCGATTTTA
*PECAM1*/CD31 (5175)	GCTGACCCTTCTGCTCTGTT	ATCTGGTGCTGAGGCTTGAC
*THBD*/THBD (7056)	ACCTTCCTCAATGCCAGTCA	AAGGAAATGACATCGGCAGC
*F3*/TF (2152)	GGCGCTTCAGGCACTACAA	TTGATTGACGGGTTTGGGTTC

## Data Availability

The dataset generated and/or analyzed in this study are available on reasonable request.

## Supplementary Materials

The following are available online at https://www.mdpi.com/article/10.3390/biomedicines9091220/s1, Figure S1: Specifity of the primer pairs employed for monitoring gene expression.
