# Supplementary material for: Endothelial Cell Activation by SARS-CoV-2 Spike S1 Protein: A Crosstalk between Endothelium and Innate Immune Cells
Source: Biomedicines. 2021 Sep 14;9(9):1220. doi: 10.3390/biomedicines9091220 (PMC8470710; doi:10.3390/biomedicines9091220)
Supplement: Supplementary file 1 [file biomedicines-09-01220-s001.zip › biomedicines-1316969-supplementary.pdf]

biomedicines

Endothelial Cell Activation by SARS-CoV-2 Spike S1 Protein: A Crosstalk between Endothelium and Innate Immune Cells

Supplementary Files

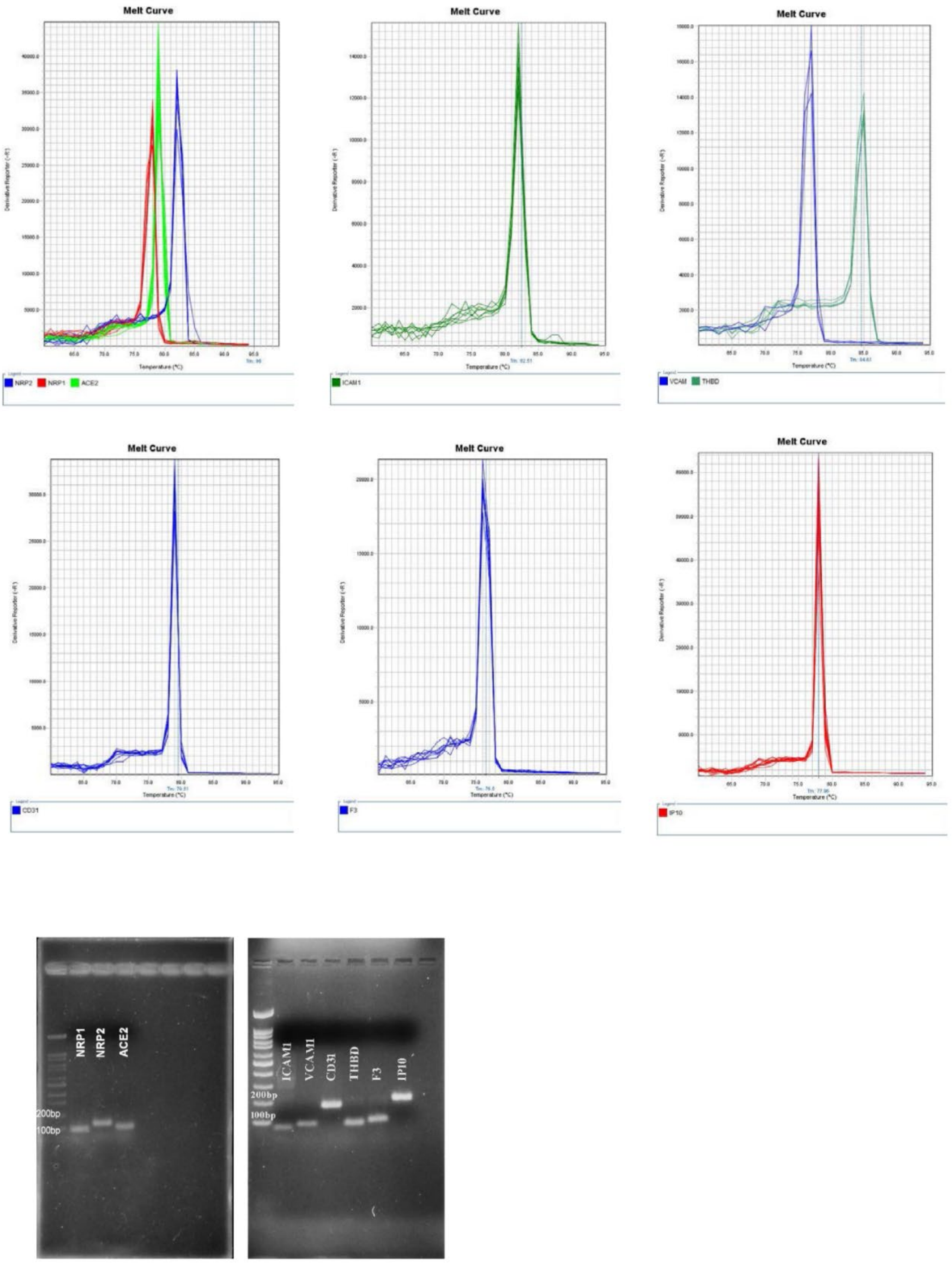

Figure S1. Specifity of the primer pairs employed for monitoring gene expression.
